# Neoadjuvant Short-Course Radiotherapy Followed by Consolidation Chemotherapy before Surgery for Treating Locally Advanced Rectal Cancer: A Systematic Review and Meta-Analysis

**DOI:** 10.3390/curroncol29050297

**Published:** 2022-05-19

**Authors:** Chun-Kai Liao, Ya-Ting Kuo, Yueh-Chen Lin, Yih-Jong Chern, Yu-Jen Hsu, Yen-Lin Yu, Jy-Ming Chiang, Pao-Shiu Hsieh, Chien-Yuh Yeh, Jeng-Fu You

**Affiliations:** 1 Division of Colon and Rectal Surgery, Department of Surgery, Chang Gung Memorial Hospital, Linkou, No. 5, Fuxing St., Guishan Dist., Taoyuan 333423, Taiwan; mr9023@cgmh.org.tw (C.-K.L.); kyu52623@cgmh.org.tw (Y.-T.K.); ld@cgmh.org.tw (Y.-C.L.); b9202063@cgmh.org.tw (Y.-J.C.); m8295@cgmh.org.tw (Y.-J.H.); jmjiang@adm.cgmh.org.tw (J.-M.C.); hsiehps@yahoo.com (P.-S.H.); chnyuh@gmail.com (C.-Y.Y.); 2 Division of Colon and Rectal Surgery, Department of Surgery, Chang Gung Memorial Hospital, Keelung Branch, No. 222, Maijin Rd., Anle Dist., Keelung City 204201, Taiwan; tomyuauk@cgmh.org.tw; 3 School of Medicine, Chang Gung University, No. 259, Wenhua 1st Road, Guishan Dist., Taoyuan 333323, Taiwan

**Keywords:** rectal cancer, short course radiotherapy, consolidation chemotherapy, pathological complete response, overall survival, disease free survival, meta-analysis

## Abstract

Neoadjuvant short course radiotherapy (SCRT) followed by consolidation chemotherapy (CCT) is an alternative treatment for locally advanced rectal cancer (LARC). We performed this systematic review and meta-analysis to explore the tumor response and oncological outcomes of this new approach compared to conventional chemoradiotherapy (CRT). An online search of the PubMed, Embase, and Cochrane Library databases was performed. This review included 7507 patients from 14 different cohorts. The pCR rate was higher with SCRT + CCT than that with CRT (RR: 1.60; 95% CI: 1.35–1.91; *p* < 0.01). SCRT + CCT provided a higher ypN0 response (RR: 1.06; 95% CI: 1.01–1.12; *p* = 0.02). There were no differences in R0 resection and positive CRM rates; however, more sphincter-preservation surgeries were performed in the SCRT + CCT arm (RR: 1.06; 95% CI: 1.01–1.11; *p* = 0.02). There was no difference in the OS and DFS between the SCRT + CCT and the CRT arms (OS: HR: 0.85, *p* = 0.07; DFS: HR: 0.88, *p* = 0.08). The compliance and toxicity were comparable between the SCRT and CRT groups. In the subgroup analysis, patients who underwent four or more cycles of CCT had better pCR and DFS events. Therefore, SCRT followed by consolidation chemotherapy might be an effective alternative treatment for LARC.

## 1. Introduction

Colorectal cancer (CRC) is the third most common cancer and the second leading cause of cancer-related deaths worldwide. According to GLOBOCAN statistics, there were over 1.9 million new CRC cases in 2020, with 39% in the rectum [1]. After the introduction of total mesorectal excision (TME) by Dr. Heald in the 1980s, the local recurrence rate of rectal cancer decreased to 4–9%, and survival reached 75–80% [2,3]. Several randomized trials have revealed that adding preoperative radiotherapy or chemoradiation improves the local recurrence rate to approximately 5% [4,5]. Therefore, surgical treatment with TME after neoadjuvant chemoradiation has become the standard treatment for locally advanced rectal cancers (LARC). In the past two decades, short-course radiotherapy (SCRT) with immediate surgery or concurrent chemoradiotherapy (CRT) with surgery four to six weeks later has been recommended by guidelines worldwide [6,7]. Despite different interpretations of treatment efficacy, several large trials have revealed the same local control and overall survival between these two approaches [8,9]. Good local control was observed in the neoadjuvant treatment of locally advanced rectal cancer. However, distal metastasis is still reported in approximately 30% of cases [9,10]. Therefore, a new treatment strategy to reduce early metastasis is urgently needed. A criticism of using SCRT with immediate surgery for LARC is that a short interval precludes the possibility of clinical tumor response and organ preservation. Recently, the Stockholm III trial showed a 10.1% tumor response rate if the interval between SCRT and surgery was increased to four to eight weeks, and the safety of delayed surgery was reported to be similar to that of in comparison with immediate surgery [11,12]. Following this result, many studies have been conducted to explore the effectiveness and feasibility of SCRT with delayed surgery and conventional CRT [11,12,13]. However, another concern is that there is no protection from chemotherapy during this long waiting interval. Therefore, it is reasonable to propose the addition of consolidation chemotherapy during this period as a new neoadjuvant treatment modality. Recently, an increasing number of studies have compared SCRT and consolidation chemotherapy with conventional CRT for the treatment of LARC [14,15,16]. The aim of this systematic review and meta-analysis is to summarize current evidence regarding neoadjuvant SCRT followed by consolidation chemotherapy before surgery for LARC, and explore the efficacy in tumor response, treatment toxicity, surgical complications, patient compliance, overall survival (OS), and disease-free survival (DFS). Furthermore, we included studies comparing SCRT with delayed surgery and conventional CRT to understand the effectiveness of consolidation chemotherapy.

## 2. Material and Methods

### 2.1. Search Strategy

This study was conducted in accordance with the guidelines of the Preferred Reporting Items for Systematic Reviews and Meta-analysis [17]. A comprehensive search was conducted using the PubMed, Embase, and Cochrane Library databases from the earliest records to 31 March 2022. The search terms were as follows: (rectal cancer or rectal neoplasm) AND (preoperative) AND (short-course radiotherapy) AND (long-course chemoradiation or long-course radiotherapy). Two reviewers independently searched the databases for eligible articles. The bibliographies of the included trials and related review articles were manually reviewed for potentially missing studies. The protocol of this systematic review was registered in PROSPERO with the registration ID CRD42021250641. 

### 2.2. PICOS-Based Inclusion and Exclusion Criteria

The inclusion criteria were designed according to the Population, Intervention, Comparison, Outcomes, and Study (PICOS) design principle and were set as follows: (1) P: patients with pathologically proven rectal cancer; (2) I: neoadjuvant SCRT, composed of a total dose of 25 Gy, followed by delayed surgery after at least four weeks, with or without consolidation chemotherapy during the waiting period; (3) C: neoadjuvant conventional CRT, composed of a total dose of 50–50.4 Gy, followed by delayed surgery after at least four weeks, with or without consolidation chemotherapy during the waiting period; (4) O: pathological complete response (pCR) rate, tumor downstaging rate, radiotherapy or chemotherapy-related grade 3/4 acute toxicity and/or late toxicity, sphincter-preservation rate, post-operative complications (anastomosis leakage, surgical site infection, and ileus), R0 resection rate, CRM free rate, overall survival, disease-free survival, local recurrence, distant metastasis, and compliance with treatment (chemotherapy or radiotherapy). Studies reporting these outcomes were included; (5) S: randomized controlled trials (RCTs) or observational studies. 

The exclusion criteria were as follows: (1) patients with synchronous metastasis during diagnosis; (2) radiotherapy not meeting the inclusion criteria; (3) delay between radiotherapy and surgery was less than four weeks; (4) lack of qualified data for extraction and analysis; (5) single-arm studies, review articles, case reports, editorials, comments, or conference abstracts. 

### 2.3. Data Extraction and Quality Assessment

Two independent reviewers examined all retrieved articles and extracted data using a predetermined form. The following information was extracted: (1) characteristics of the study: author name, publication year, study period, location, and study type; (2) characteristics of the study cohort: patient number, age, sex, stage, tumor location, intervention (including RT dose, chemotherapy regimen, surgery type, and adjuvant chemotherapy), and follow-up time; (3) outcomes: pCR rate, downstaging rate, sphincter preservation rate, R0 resection rate, grade 3/4 acute toxicity (during neoadjuvant treatment), postoperative grade 3/4 complications, late toxicity, compliance with treatment (including RT and chemotherapy), local recurrence, distant metastasis, OS, and DFS. Inconsistencies between the two reviewers were resolved through discussions. The risk of bias was assessed by two reviewers and all discrepancies were resolved after consensus with the corresponding author. We used the Cochrane risk of bias assessment tool 2.0 to evaluate the quality of RCTs. The tool contained five domains, including bias in the randomization process, deviations from the intended interventions, missing outcome data, bias in the measurement of the outcomes, and selection of the reported result. Each domain was assessed as low and high risk of bias, or as that having some concern [18]. The quality of the observational studies was assessed using the Newcastle–Ottawa Quality Assessment Scale (NOS). NOS contains nine items in three categories: participant selection (four items), comparability (two items), and exposure (three items). A study can be scored a maximum of one point for each item in the selection and exposure domains and two points for the comparability domain [19]. A study with a NOS score of seven or higher was defined as a high-quality study.

### 2.4. Statistical Analysis

All statistical analyses were performed using Review Manager (RevMan version 5.4) from the Cochrane Collaboration. The categorized variables were calculated using the Mantel-Haenszel method and presented as risk ratios (RRs). Survival analysis was calculated using the inverse variance method using the hazard ratio (HR) and 95% confidence interval (CI) extracted directly from the original studies while reporting survival analysis of OS or DFS. We also analyzed OS, DFS, local recurrence (LR), and distant metastasis (DM) at a reported time point using the Mantel-Haenszel method and presented them as risk ratios (RRs). Heterogeneity between the studies was determined using the Cochran Q-test and I^2^ statistics. Considerable heterogeneity was defined as I^2^ ≥ 50%. A random-effects model was used in this meta-analysis owing to a difference in the study design and the enrolled participants. A subgroup analysis was performed to investigate the pooled effect with or without consolidation chemotherapy after SCRT and cycles of consolidation chemotherapy. 

### 2.5. Interpretation

The SCRT group (with or without CCT) was set as the experimental arm and the CRT group was set as the control arm in this meta-analysis. The comparisons measured the ratio of experimental arm versus control arm. Thus, when the outcome represented as RRs, the value greater than one means a higher rate of events in the SCRT group. When the outcome is represented as HRs, which was used in the survival analysis, a value greater than one means that the survival is worse in the SCRT group. 

## 3. Results

### 3.1. Study Selection 

**Figure 1:**
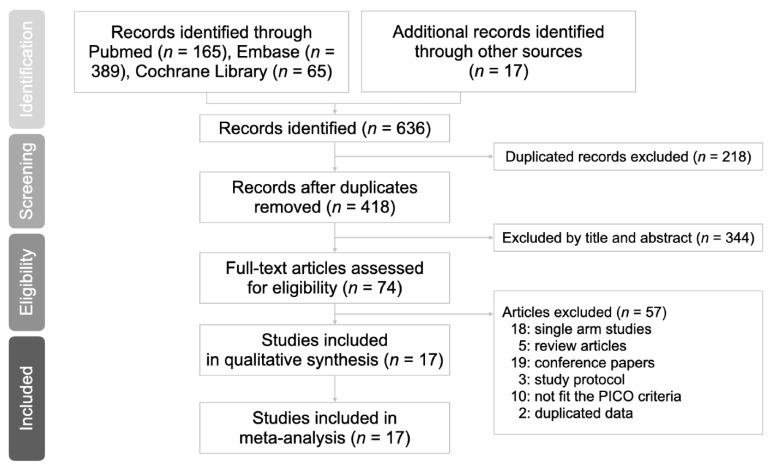
Preferred reporting items for systematic reviews and meta-analyses flow diagram to search and identify included studies.

A total of 636 articles were identified based on the online databases and manual searches, of which 218 duplicate records were removed. After reviewing the titles and abstracts of the articles, 344 were removed. After assessing the full text of the remaining 74 articles, 57 were excluded owing to 18 single-arm studies, five reviews, 19 conference papers, three study protocols, 10 had incomplete data, and two had duplicated data. Finally, 17 eligible studies were included in the meta-analysis. The flow diagram is shown in Figure 1.

### 3.2. Characteristics of the Included Study

Seventeen studies enrolling 7507 rectal cancer patients from 13 cohorts were included in this meta-analysis [13,14,15,16,20,21,22,23,24,25,26,27,28,29,30,31,32]. Of these, two studies reported different parameters of the same cohort [28,29], four studies reported survival outcomes at different time points [13,14,20,24], and one study reported an expanded enrollment including the preliminary cohort [31]. All studies were published between 2015 and 2022; of these, ten were RCTs, two were prospective studies, and five were retrospective studies. In summary, four cohorts from five studies compared SCRT with delayed surgery (*n* = 803) and conventional CRT (*n* = 4075) [13,20,21,22,23], and nine cohorts from 12 studies compared SCRT followed by consolidation chemotherapy with delayed surgery (*n* = 1452) and conventional CRT (*n* = 1452) [14,15,16,24,25,26,27,28,29,30,31,32]. The characteristics of the included studies are summarized in Table 1.

### 3.3. Quality Analysis

**Figure 2:**
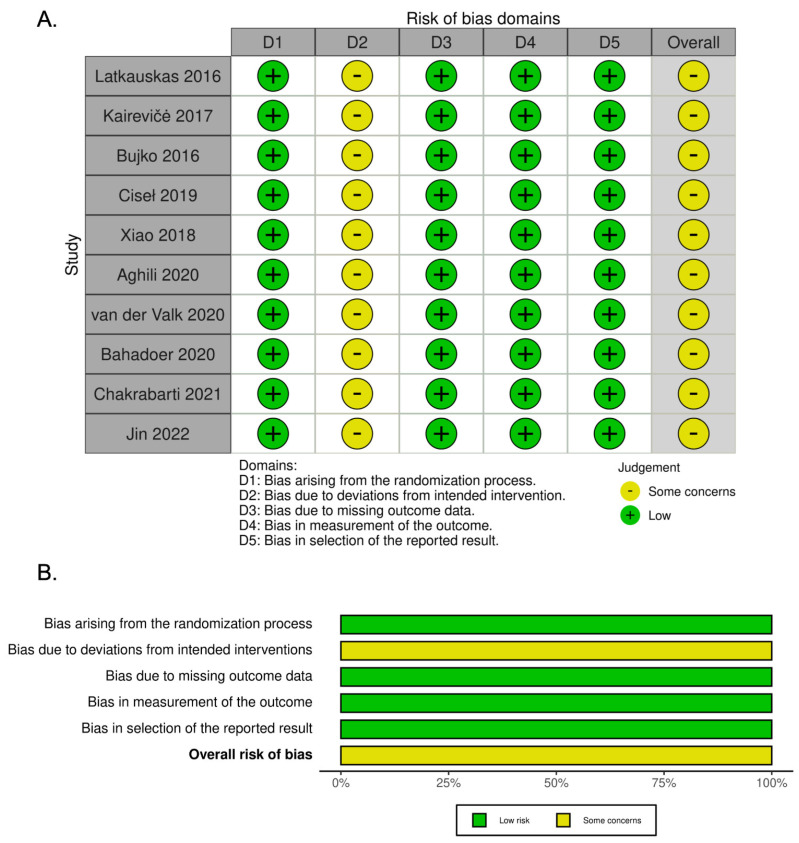
Risk of bias (**A**) assessment and (**B**) summary of the RCTs.

All of the included RCTs in this study reported the randomization process; however, patients were informed regarding their treatment plan at allocation because of the difficulty in keeping the radiotherapy regimen a secret between the patients and researchers. However, the above restriction did not affect the outcome assessment. Considering deviations from the intended interventions, there was a disproportion regarding completion of the intended adjuvant chemotherapy. In most studies, adjuvant chemotherapy was optional after CRT; thus, there was heterogeneity in the control group. The patients with or without adjuvant chemotherapy may influence the outcomes. As a result, “some concerns” was graded for all the RCTs in “deviations from the intended interventions” section. The risk of bias graphs and summary is shown in Figure 2. The quality of the seven cohort studies was evaluated using the Newcastle-Ottawa Scale (NOS). As shown in Table 2, the NOS scores of the included studies ranged from 7 to 9; therefore, they were regarded as being of high-quality.

### 3.4. Tumor Response and pCR Rate

**Figure 3:**
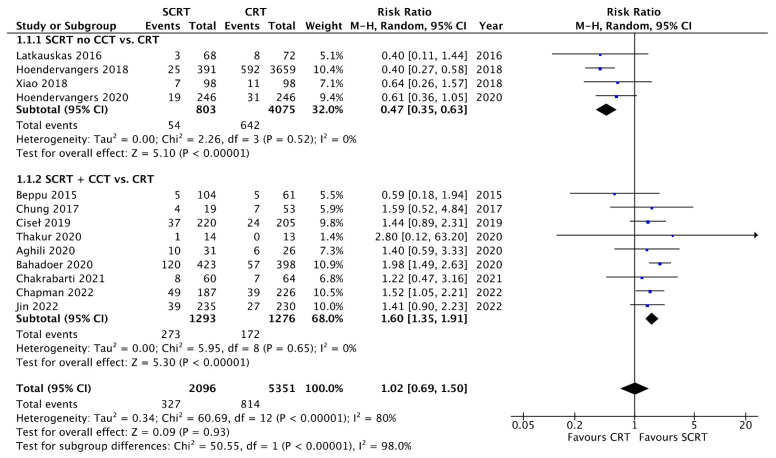
Forest plot of pathological complete response (pCR).

The details of the outcomes are summarized in Table 3. All studies provided data for analyzing the pCR rates. As shown in Figure 3, SCRT followed by consolidation chemotherapy provided a higher pCR rate compared to that with conventional CRT (RR: 1.60; 95% CI: 1.35–1.91, *p* < 0.01). In contrast, SCRT with delayed surgery and no consolidation chemotherapy had a lower pCR rate compared to that with conventional CRT (RR: 0.47; 95% CI: 0.35–0.63; *p* < 0.01). Regarding tumor downstaging, nine studies reported the comparison results, and there was no difference between SCRT + CCT and conventional CRT (RR: 1.04; 95% CI: 0.75–1.45; *p* = 0.81). However, lower downstaging was observed in the SCRT with delayed surgery group (RR: 0.82; 95% CI: 0.74–0.91; *p* < 0.01) (Figure S1). Regarding the ypT3-4 response, SCRT with CCT showed a better response than that with conventional CRT (RR: 0.87; 95% CI: 0.74–1.01; *p* = 0.07) (Figure S2). Regarding the ypN0 response, SCRT with CCT was better than conventional CRT (RR: 1.06; 95% CI: 1.01–1.12; *p* = 0.02) (Figure S3).

### 3.5. Overall Survival and Disease-Free Survival

**Figure 4:**
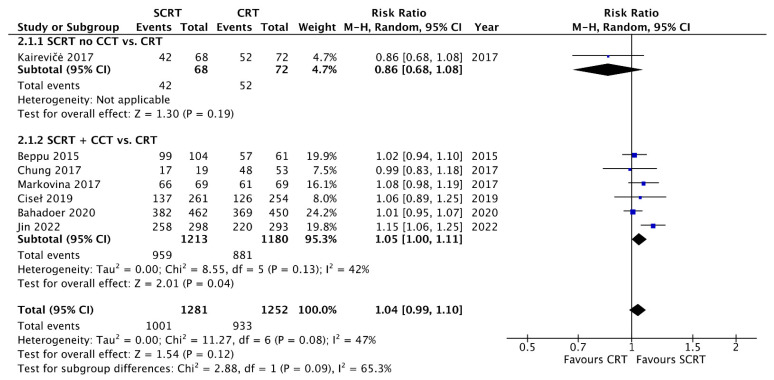
Forest plot of overall survival (OS) events by Risk ratio (RR).

**Figure 5:**
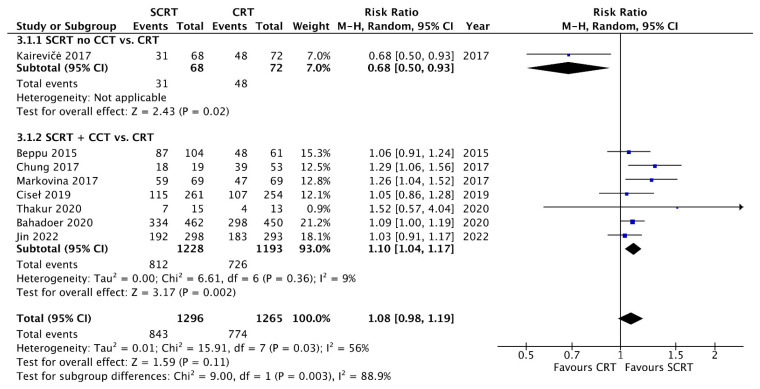
Forest plot of disease-free survival (DFS) events by Risk ratio (RR).

**Figure 6:**
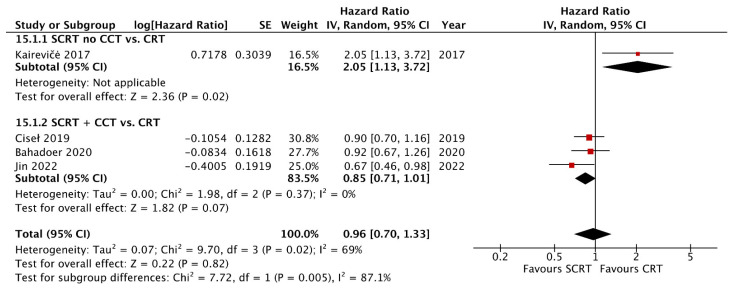
Forest plot of the overall survival (OS) by Hazard ratio (HR).

**Figure 7:**
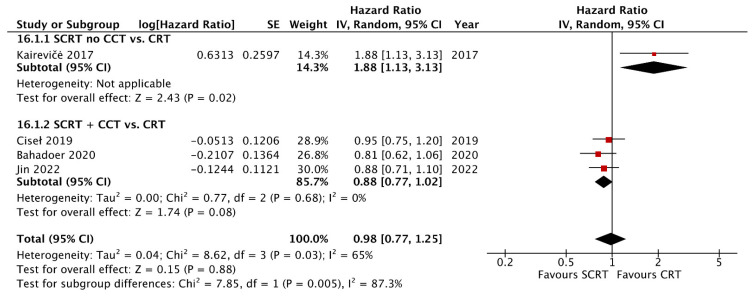
Forest plot of the disease free survival (DFS) by Hazard ratio (HR).

A total of seven studies including 2533 patients reported the OS data at a fixed time point for assessment with a follow-up interval between 25 months and seven years. There was no statistical difference between SCRT with delayed surgery (RR: 0.86, 95% CI: 0.68–1.08, *p* = 0.19) and conventional CRT, but a superior OS was observed in the SCRT + CCT (RR: 1.05; 95% CI: 1.00–1.11; *p* = 0.04) arm by using RRs as the estimation (Figure 4). Regarding DFS, eight studies, including 2561 patients, reported DFS data at a fixed time point. SCRT + CCT led to significantly better DFS compared to conventional CRT (RR: 1.10; 95% CI: 1.04–1.17; *p* = 0.002). In contrast, SCRT with delayed surgery had a worse DFS compared to that with conventional CRT (RR: 0.68; 95% CI: 0.50–0.93; *p* = 0.02) (Figure 5). Regarding the survival rate by hazard ratio, which was available and analyzed only in four RCTs, one in the SCRT with delayed surgery arm and three in the SCRT + CCT arm. The OS and DFS were superior in the conventional CRT arm to those in the SCRT with delayed surgery. A marginally significant effect favoring the SCRT with the CCT arm was observed. The OS (HR: 0.85; 95% CI: 0.71–1.01; *p* = 0.07) and DFS (HR: 0.88; 95% CI: 0.77–1.012; *p* = 0.08) were estimated by combining the three RCTs (Figure 6 and Figure 7).

Data on the incidence of local recurrence and distant metastasis were reported in eight and seven studies, respectively. There was no statistical difference in LR between SCRT + CCT and conventional CRT (RR: 1.12; 95% CI: 0.91–1.37; *p* = 0.26) and SCRT with delayed surgery and conventional CRT (RR: 0.85; 95% CI: 0.24–3.02; *p* = 0.80) (Figure S4). Regarding the incidences of DM, there was no statistical difference between SCRT + CCT and conventional CRT (RR: 0.86; 95% CI: 0.68–1.07; *p* = 0.18) (Figure S5).

### 3.6. R0 Resection, Negative CRM, Sphincter-Preservation Rate, and Postoperative Complications

**Figure 8:**
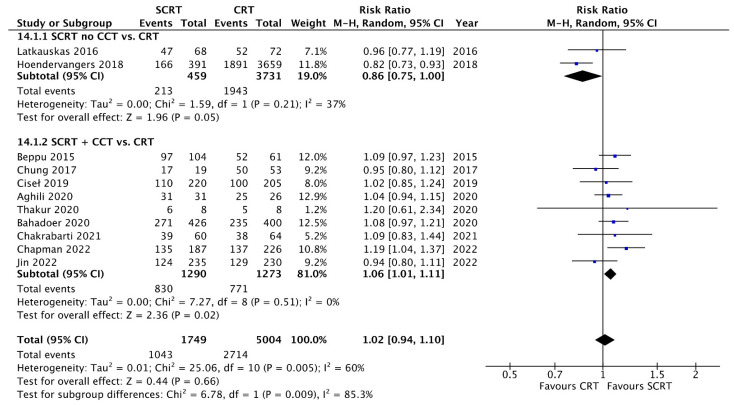
Forest plot of the sphincter-preservation rate.

Ten studies reported R0 resection rates for assessment, and there was no statistical difference between the SCRT and conventional CRT arms regarding R0 resection rates, either with delayed surgery (RR: 1.01; 95% CI: 0.93–1.09; *p* = 0.87) or consolidation chemotherapy (RR: 1.02; 95% CI: 0.99–1.05; *p* = 0.11) (Figure S6). The negative CRM rates were also similar between the SCRT and conventional CRT arms (RR: 1.00; 95% CI: 0.98–1.02; *p* = 0.90) (Figure S7). Regarding sphincter preservation rates, 11 studies enrolling 6753 patients offered data for assessment. SCRT + CCT had a superior sphincter preservation rate compared to conventional CRT (RR: 1.06; 95% CI: 1.01–1.11; *p* = 0.02). In contrast, SCRT with delayed surgery had a lower sphincter preservation rate compared to the CRT arm (RR: 0.86; 95% CI: 0.75–1.00; *p* = 0.05) (Figure 8). There was no difference in postoperative complications between SCRT with delayed surgery or SCRT + CCT and conventional CRT. However, a borderline significance of RR was observed when the two models of SCRT were combined (RR: 1.10; 95% CI: 1.00–1.21; *p* = 0.06) (Figure S8).

### 3.7. Acute Toxicity, Late Toxicity, and Compliance of Treatment

Grade 3+ acute toxicity was reported by nine studies and no difference was observed between the SCRT + CCT and CRT arms (RR: 1.30; 95% CI: 0.91–1.85; *p* = 0.15), in contrast to a superior outcome in the SCRT with delayed surgery arm (RR: 0.19; 95% CI: 0.08–0.48; *p* < 0.001) (Figure S9). The incidences of grade 3+ late toxicity were only available in four studies, and the SCRT + CCT arm was worse compared to the CRT arm (RR: 1.32; 95% CI: 1.08–1.62; *p* = 0.008) (Figure S10A). The overall compliance with radiotherapy was better for SCRT with a 100% completion rate reported in five studies, in contrast to 1.8% to 7.7% of patients requiring dose reduction in the CRT arm. No difference in chemotherapy dose reduction was observed between the SCRT + CCT and CRT arms (RR: 1.12; 95% CI: 0.31–3.96; *p* = 0.87) (Figure S10B).

### 3.8. Subgroup Analysis according to the Consolidation Chemotherapy Cycle 

**Figure 9:**
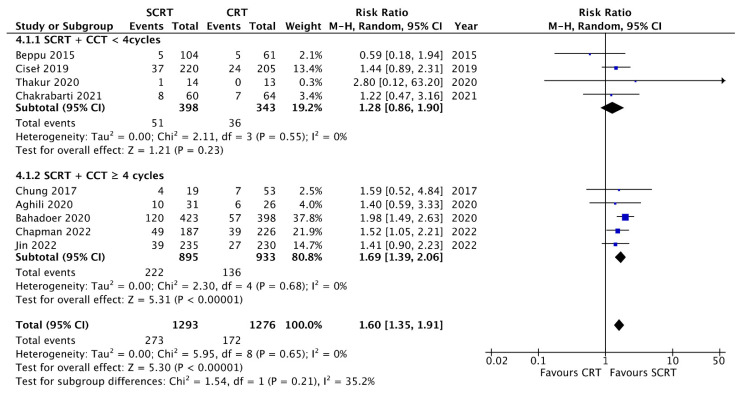
Forest plot for subgroup analysis of the pathological complete response (pCR) according to consolidation chemotherapy cycles.

**Figure 10:**
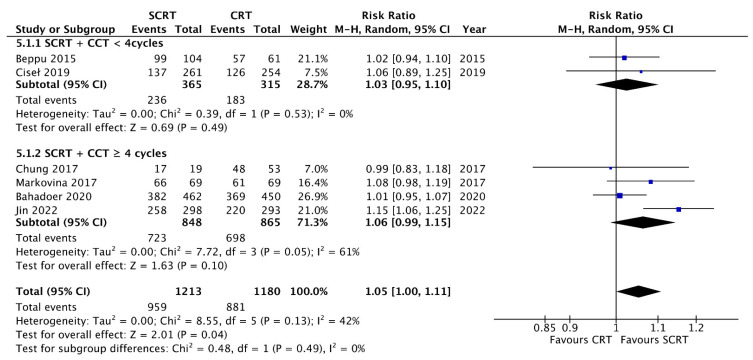
Forest plot for subgroup analysis of the overall survival (OS) events according to consolidation chemotherapy cycles.

**Figure 11:**
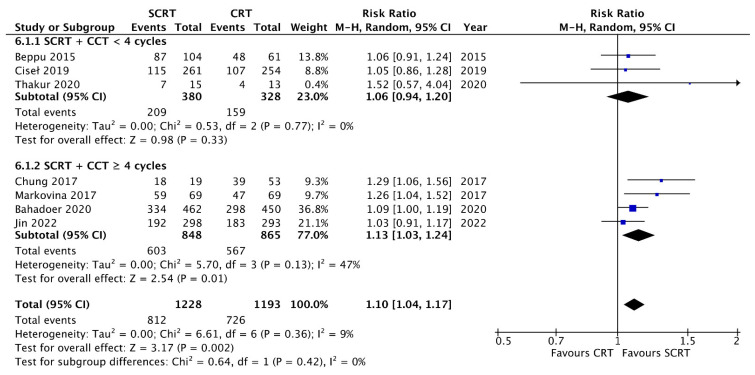
Forest plot for subgroup analysis of the disease free survival (DFS) events according to consolidation chemotherapy cycles.

The subgroup analysis showed a significantly better pCR rate with SCRT followed by at least four cycles of consolidation chemotherapy compared to the CRT arm (RR: 1.93; 95% CI: 1.52–2.45; *p* < 0.01). In contrast, no difference was observed in the SCRT arm as consolidation chemotherapy was administered in less than four cycles (RR: 1.32; 95% CI: 0.83–2.08; *p* = 0.24) (Figure 9). There was no difference between the SCRT and CRT arms regarding CCT cycles on OS events (Figure 10); however, significantly better DFS events were observed in CCT cycles ≥ 4 subgroups (RR: 1.13; 95% CI: 1.03–1.24; *p* = 0.01), and no difference was observed in the CRT arm with the CCT cycles < 4 subgroups (RR: 1.06; 95% CI: 0.94–1.20; *p* = 0.33) (Figure 11).

## 4. Discussion

This meta-analysis demonstrated that treating LARC with SCRT followed by consolidation chemotherapy provides better pCR rates, results in more ypN0 status from positive pre-treatment lymph nodes, and increases sphincter preservation surgery. Moreover, improved tumor downstaging status owing to the lesser presentation of the ypT3-4 tumor after surgery and a trend of reduced distant metastasis were observed; the OS and DFS were comparable to those in patients who underwent conventional CRT although there was insufficient evidence to draw a conclusion regarding the R0 resection rates, treatment toxicity, and complications. Although increasing pCR rates were also observed after SCRT with delayed surgery, the above parameters did not improve with an increased time interval before surgery compared to the conventional CRT. 

The improved pCR rate might be attributed to the prolonged interval between radiotherapy, surgery, and the addition of systemic chemotherapy during this period. Regarding traditional neoadjuvant treatment, a criticism for applying SCRT with immediate surgery in LARC is that the short interval precludes the possibility of clinical tumor response. In the Stockholm III trial, the experimental arm showed an increasing pCR rate if surgery was delayed for four to eight weeks after SCRT, and it could reach 11.8%, compared to 2.1% in the SCRT with the immediate surgery group [11]. According to previous studies, an increasing pathological response was observed after SCRT with delayed surgery; however, the pCR rate was not greater than after conventional CRT [13,20,21,22,23]. Moreover, the pooled results in this meta-analysis showed a significantly lower pCR rate compared to conventional CRT in patients who underwent SCRT followed by delayed surgery without consolidation chemotherapy. Therefore, simply extending the interval between SCRT and surgery cannot replace conventional CRT in the treatment of LARC. 

Previous studies have shown better long-term OS and DFS in patients with tumor regression or pCR after neoadjuvant treatment for LARC [33,34]. Adding systemic chemotherapy to neoadjuvant treatment was one proposal for improving tumor response. In a prospective phase II trial conducted by Garcia-Aguilar et al., the effect on tumor response with additional mFOLFOX-6 cycles between conventional CRT and surgery was compared. There was an obvious improvement in tumor response with increasing chemotherapy cycles, with a 25% pCR rate in two cycles of the mFOLFOX-6 group and up to 38% in six cycles of the mFOLFOX-6 group [35]. This finding indicates the potential for tumor shrinkage if systemic chemotherapy is added to neoadjuvant treatment. Many studies have focused on the feasibility and safety of adding consolidation or induction chemotherapy to typical (chemo) radiotherapy. A better pCR rate and tumor response, including turning more lymph node-negative and having a less ypT3-4 stage, were observed in this meta-analysis, revealing the feasibility of SCRT with consolidation chemotherapy in treating LARC. With a better tumor response, more patients can undergo sphincter-preservation surgery with no increase in the complication rate compared to conventional CRT. The improved pCR rate and sphincter preservation surgery also indicate the potential of organ preservation treatment, including the “watch and wait” strategy [36,37]. 

Regarding the oncological outcome, this meta-analysis showed no difference in OS between SCRT and CRT, regardless of consolidation chemotherapy using an event-to-patient ratio. However, diverse DFS rates were observed. The pooled DFS events in SCRT followed by consolidation chemotherapy was better compared to conventional CRT. Because the above outcomes contain data from both observational studies and RCTs, the estimation may have a higher risk of bias and we should pay attention to these results. Considering the OS and DFS using the time-to-events ratio, only four RCTs provided the HRs for analysis. The study conducted by Kairevičė et al. revealed poorer OS and DFS in SCRT with a delayed surgery arm compared to that in the conventional CRT arm. This was foreseeable because only the CRT arm participants received adjuvant chemotherapy after surgery [20]. Three RCTs, from the Polish II, RAPIDO, and STELLAR trials, compared SCRT with consolidation chemotherapy and conventional CRT [14,29,32]. Although no significant difference in the pooled OS and DFS was observed, a trend favoring the SCRT + CCT arm was observed. However, these results should be interpreted cautiously owing to some obvious heterogeneities within these studies. First, the administration and completion of adjuvant chemotherapy varied across studies. Optional adjuvant chemotherapy was used in both arms in the Polish II trial in contrast to adjuvant chemotherapy usage only in the conventional CRT arm in the RAPIDO trial and in both arms in the STELLAR trial. In the Polish II trial, oxaliplatin-based chemotherapy was given in only 15% of the experimental group and 11% of the control group [14]. The completion rate of the scheduled dose of adjuvant chemotherapy was reported to be 47% in the RAPIDO trial and 48% in the STELLAR trial [28,32]. A recent study explored that adjuvant chemotherapy may improve the DFS in the RAPIDO trial by sensitivity analysis and indicated the decision of making adjuvant chemotherapy optional may have biased the results in favor of the experimental arm [38]. Another heterogeneity we should pay attention to is that the clinical stage of the enrolled patients differed among studies. The RAPIDO trial enrolled patients with mostly bulky tumors compared to the other two trials. The disparity regarding the trial design should be considered while examining the results of this meta-analysis.

The distinct chemotherapy regimens between the SCRT and CRT arms were also confounding factors influencing the oncological outcomes. Of the nine cohorts included for analysis, a combination of oxaliplatin and 5-FU based regiments was administered in seven cohorts and a single 5-FU based chemotherapy was used in the remaining two cohorts after SCRT compared to most studies that applied a single 5-FU agent in CRT. Several previous studies showed the effectiveness of adding oxaliplatin in neoadjuvant chemotherapy considering oncological outcomes. Marco et al. described the effect of two, four, or six cycles of mFOLFOX6 consolidation chemotherapy between CRT and TME surgery. There was no difference in OS; however, a significantly better DFS was found in patients who underwent CRT and consolidation chemotherapy [39]. Another study conducted by Liang et al., showed an improved pCR/near pCR rate (32.80% vs. 16.25%; *p* = 0.015) and 3-year DFS (85.48% vs. 56.54%; *p* = 0.036) in patients who received consolidation chemotherapy during the resting period between CRT and surgery [40]. The effect of adding oxaliplatin to a single agent, 5-FU, in neoadjuvant CRT was elucidated in a systematic review. Thavaneswaran et al., found that the combination regimen is superior to a single 5-FU agent regimen in terms of three-year DFS (HR: 0.79; 95% CI: 0.68–0.93; *p* = 0.004), LR (HR: 0.75; 95% CI: 0.58–0.97; *p* = 0.03), and DM rate (HR: 0.78; 95% CI: 0.64–0.96; *p* = 0.02); however, it was not superior to OS (HR: 0.89; 95% CI: 0.75–1.06; *p* = 0.19) [41]. 

Considering analysis of recurrence patterns, the pooled results were not significantly different between the SCRT with the CCT arm and CRT arms with regard to either local recurrence or distant metastasis in this meta-analysis. However, a trend towards more local recurrence events and less distant metastasis events in SCRT with the CCT arm was observed. In the updated five-year results of the RAPIDO trial, the author demonstrated increasing cumulative local recurrence rates (10% vs. 5%; *p* = 0.010) and decreasing distant metastasis rates (23% vs. 31%, HR 0.72; *p* = 0.011) in the experimental arm [42]. This indicates that a longer follow up time is warranted for exploring the effectiveness of the new treatment modality and for accurately interpreting the current data. 

A meta-analysis showed a reduction in LR and DM and an increase in DFS if an oxaliplatin-based adjuvant chemotherapy was administered after CRT and TME surgery for rectal cancer patients than those that used a fluorouracil-based chemotherapy [43]. However, the OS did not differ between the two regimens. Thus, trimodal therapy, incorporating neoadjuvant treatment, TME, and adjuvant chemotherapy is now considered the standard treatment model for LARC. However, compliance with adjuvant chemotherapy is diverse across trials and has been reported to be between 43 and 73% [44]. Relatively improved compliance was observed in neoadjuvant treatment, another new treatment model, by shifting the adjuvant systemic chemotherapy before surgery. It was proposed and named "total neoadjuvant treatment" (TNT). Diefenhardt et al., conducted a post-hoc analysis of the CAO/ARO/AIO-04 trial and found that treatment adherence was associated with the three-year DFS. In the CRT with fluorouracil-oxaliplatin regimen arm, patients who underwent full-dose scheduled CRT had better DFS compared to those who underwent near-complete CRT (HR: 1.501; 95% CI: 0.980–2.299; *p* = 0.06) and those who underwent reduced-dose CRT (HR, 1.724; 95% CI, 1.144–2.596; *p* = 0.009). The above findings indicate the importance of treatment designs concerning the treatment dose, schedule, and any supportive strategies to facilitate good adherence [45], and support the rationale of using TNT in treating LARC.

In this meta-analysis, we found that compliance was good in the SCRT followed by the chemotherapy group. A 100% completion rate for SCRT was reported in each study, mainly because of the short treatment time required. Owing to the shorter treatment time, SCRT can increase patient convenience and reduce medical costs and is favored in many countries [46]. Compliance with consolidation chemotherapy depends on the regimen. In this meta-analysis, the pooled data regarding the incidence of reduced chemotherapy doses did not differ between the SCRT and CRT arms. However, the results showed high heterogeneity across studies, and careful interpretation should be performed. For grade 3+ acute toxicity, this meta-analysis showed no difference between SCRT followed by consolidation chemotherapy and the conventional CRT arm. However, the results varied across studies. The between-study differences may be attributed to the different chemotherapy regimens and cycles used. As previous studies showed a better tumor response and DFS with the addition of consolidation mFOLFOX-6 before surgery, a synchronized increase in the incidence of acute toxicity was also observed. In a meta-analysis by Thavaneswaran et al., acute grade 3/4 toxicity was common in the combination agent group (HR: 1.67; 95% CI: 1.21–2.31; *p* = 0.002), and radiotherapy compliance was lower compared to the single-agent group (HR: 0.41; 95% CI: 0.26–0.67; *p* = 0.0003) [41]. In brief, the acute toxicity of consolidation chemotherapy increased with the cycles of the combination regimen administered, resulting in poor compliance with CRT. If we use SCRT instead of long course radiotherapy, we can minimize its influence on radiation compliance and achieve good treatment adherence. The last toxicity was less mentioned, and the assessed time varied in previous studies; therefore, the results that SCRT followed by consolidation chemotherapy leads to more late complications should be expounded carefully. 

Currently, there is no consensus regarding the cycles of consolidation chemotherapy that should be administered before surgery. In the subgroup analysis of chemotherapy cycles, we found a superior pCR rate and DFS events if four or more cycles of chemotherapy were applied. This finding is similar to that in previous studies that incorporated consolidation chemotherapy and CRT, which showed a superior pCR rate in patients who underwent six cycles of chemotherapy compared to those who underwent four or two cycles of chemotherapy [33,36]. Moreover, there is no consensus on whether induction or consolidation chemotherapy should be combined with radiotherapy. In the phase III PRODIGE 23 trial, an intensive chemotherapy regimen, FOLFIRINOX, was administered as induction chemotherapy before CRT and surgery. This experimental arm showed a 28% pCR rate and improved DFS compared to conventional CRT. Moreover, the incidence of neurotoxicity was lower in the adjuvant chemotherapy period compared to the control group. This finding convinced us that induction chemotherapy is more efficient and better tolerated compared to conventional CRT with adjuvant chemotherapy [47]. The phase III RAPIDO trial, which compared SCRT followed by consolidation mFOLFOX-6/CAPOX chemotherapy to conventional CRT, had a similar pCR rate (28%) as the PRODIGE 23 trial. Although considerable preoperative toxicity was noted compared to CRT, there was no difference in surgical complications [28,29].

Recently, the CAO/ARO/AIO-12 trial, which compared induction or consolidation chemotherapy with conventional CRT, reported a higher pCR rate in the consolidation group (25% vs. 17%; *p* < 0.001). Treatment toxicity and patient compliance were also more favorable in the consolidation group [48]. The long-term results showed no differences in OS, DFS, and incidences of LR and DM in both groups [49]. Therefore, the authors concluded that CRT followed by consolidation chemotherapy is the preferred TNT sequence if organ preservation is a priority. In a recent meta-analysis comparing TNT and standard therapy in LARC, TNT was associated with a high change in pCR (odds ratio: 2.44; 95% CI: 1.99–2.98; *p* < 0.001) [50]. However, the enrolled studies included chemotherapy combined with neoadjuvant SCRT and conventional CRT. There is still no evidence regarding which type of radiotherapy is better for TNT because no study has investigated a direct comparison. This meta-analysis focuses on the use of SCRT followed by consolidation chemotherapy because of several advantages regarding the use of SCRT. First, the shorter interval of treatment was convenient for patients and achieved good compliance. Second, medical costs are lower because of the shorter treatment duration. A randomized phase III trial, ACO/ARO/AIO-18.1 conducted by the German Rectal Cancer Study Group was proposed recently that compared SCRT and CRT, followed by consolidation chemotherapy and selective organ preservation for MRI-defined intermediate and high-risk rectal cancer patients [51]. The results of this RCT may answer the above questions. 

There are some limitations to this study. First, the number of participants in most studies was small, and only three large RCTs included over 100 patients in each arm. Second, the regimen and cycles of consolidation chemotherapy varied across studies, limiting a conclusive result regarding which regimen is better. Third, although most studies enrolled patients with stage II-III rectal cancer, clinical heterogeneity remained, as tumor location varied across each study. Furthermore, studies that included patients that were at the advanced stage of the disease could also affect the results. Finally, the administration and completion of adjuvant chemotherapy varied across studies, even in the RCTs. This deviation in intervention may lead to a favorable result in the experimental arm. Moreover, survival events were reported at various follow-up times (25 months to seven years). Thus, our results should be interpreted carefully because the current data are not robust enough to set a strong conclusion. However, we believe that this meta-analysis provides an overview of the current evidence regarding the use of SCRT with delayed surgery and consolidation chemotherapy.

## 5. Conclusions

SCRT with delayed surgery can provide tumor control comparable to conventional CRT. Moreover, SCRT followed by consolidation chemotherapy can improve tumor downstaging and induce more pCR, which may help in sphincter-preservation surgery. However, DFS, OS, toxicity, postoperative complications, and treatment compliance of the individuals were similar to those who underwent conventional CRT. Therefore, SCRT followed by consolidation chemotherapy can be considered as an alternative treatment for LARC. However, individuals should be treated according to the tumor pattern and their compliance.

## Figures and Tables

**Table 1:** Summary of the characteristics of the included studies.

Study	Enrollment Years	Study Design	Staging	Intervention	Patient Numbers (male%)	Age (Years)	RT Dose	CT Regimen	Interval (Weeks)	ACT Regimen (Completion%)	Follow Up Time (Months)
Latkauskas 2016 [13]	2007–2013	RCTs	II–III	SCRT	68 (63%)	65.6	25	No	6–8	no	39.7
				CRT	72 (68%)	63.1	50	Infusion 5FU/LV	6–8	Infusion 5FU/LV (72%)	
Kairevičė 2017 [20]	2007–2013	RCTs	II–III	SCRT	68 (63%)	65.6	25	No	6–8	no	60.5
				CRT	72 (68%)	63.1	50	Infusion 5FU/LV	6–8	Infusion 5FU/LV (72%)	
Bujko 2016 [14]	2008–2014	RCTs	cT3–4	SCRT	261 (70%)	60	25	FOLFOX × 3	12	oxaliplatin-based (15%)	35
				CRT	254 (66%)	59	50.4	Infusion 5FU/LV + Oxaliplatin × 2	6	oxaliplatin-based (11%)	
Ciseł 2019 [24]	2008–2014	RCTs	cT3–4	SCRT	261 (70%)	60	25	FOLFOX × 3	12	No	7 years
				CRT	254 (66%)	59	50.4	Infusion 5FU/LV + Oxaliplatin × 2	6	No	
Beppu 2015 [25]	2007–2013	retrospective	cT3	SCRT	106 (68%)	61	25	S1 × 10days	4	Oral 5-FU (83.7%)	44
				CRT	61 (73%)	63	45	S1 + CPT-11	6–10	Oral 5-FU (77%)	45
Chung 2017 [16]	2010–2015	retrospective	II–III	SCRT	19 (52%)	72	25	Infusion 5FU/LV × 4	8	Infusion 5 FU/LV (57.9%)	25
				CRT	53 (71%)	72	50.4	Infusion 5FU/LV	8	Infusion 5FU/LV or Xeloda (73.6%)	25
Markovina 2017 [15]	2009–2012	Phase II trial	cT3–4	SCRT	69 (71%)	57.2	25	FOLFOX × 6	4–9	NA (86%)	49.4
				CRT	69 (67%)	56.6	40–48	5-FU or capecitabine	6–8	FOLFOX (100%)	54.3
Chapman 2022 [31]	2009–2018	retrospectivr	II–III	SCRT	187 (62%)	NA	25	FOLFOX 2–6 months	4	NA (60.1%)	28.3
				CRT	226 (67%)	NA	45–55	5-FU	4	Oxalipaltin + 5 FU (82.3%)	41.6
Hoendervangers 2018 [21]	2008–2014	retrospective	II–III	SCRT	391 (50%)	76	25	No	9.1	NA	2.4 years
				CRT	3659 (64%)	63	45–50	capecitabine	9.4	NA	3.2 years
Xiao 2018 [22]	2014–2017	RCTs	II–III	SCRT	98 (48%)	59.6	25	No	6–8	No	NA
				CRT	98 (58%)	59.0	50	Infusion 5FU/LV	6–8	5FU/LV	
Aghili 2020 [26]	2016–2020	RCTs	II–III	SCRT	33 (55%)	56	25	Xelox × 3–4	8	NA	6
				CRT	27 (62%)	53	50–50.4	Xeloda + Xelox	8	NA	6
Hoendervangers 2020 [23]	2014–2017	retrospective	II–III	SCRT	246 (58%)	76.7	25	No	11	NA	NA
				CRT	246 (66%)	75.9	45–50	capecitabine	11	NA	
Thakur 2020 [27]	2015–2016	Prospective	cT3–4	SCRT	15	NA	25	Capecitabine + Oxaliplatin × 2	4–6	NA	22.6
				CRT	13	NA	45	capecitabine	4–6	NA	
van der Valk 2020 [28]	2011–2016	RCTs	* cT4, cN2	SCRT	460 (65%)	61	25	FOLFOX × 9 or Capox × 6	2–4	no	NA
				CRT	441 (69%)	61	50.4	capecitabine	6–8	CAPOX or FOLFOX	
Bahadoer 2020 [29]	2011–2016	RCTs	* cT4, cN2	SCRT	462 (65%)	62	25	FOLFOX × 9 or Capox × 6	2–4	no	4.6 years
				CRT	450 (69%)	62	50.4	capecitabine	6–8	CAPOX or FOLFOX (47%)	
Chakrabarti 2021 [30]	2017–2019	RCTs	II–III	SCRT	69 (67%)	42	25	Capox × 2	6–8	CAPOX (85%)	NA
				CRT	71 (66%)	43	50.4	capecitabine	8–12	CAPOX (52%)	
Jin 2022 [32]	2015–2018	RCTs	II–III	SCRT	302 (72%)	55	25	CAPOX × 4	6–8	CAPOX ×2 (60%)	35
				CRT	297 (70%)	56	50	capecitabine	6–8	CAPOX ×6 (48%)	

* cT4a/b, cN2, EMVI, mesorectal fascia involvement, and LLN+. NA: “Not avalible”.

**Table 2:** Scores of the observational studies according to the Newcastle-Ottawa Scale.

	Selection				Comparability	Exposure			
Author	Representativeness of the Exposed Cohort	Selection of the Nonexposed Cohort	Ascertainment of Exposure	Demonstration That the Outcome of Interest Was Not Present at the Start of the Study	Comparability of Cohorts Based on the Design or Analysis	Assessment of Outcome	Was Follow-Up Long Enough for Outcomes to Occur	Adequacy of the Follow-Up of Cohorts	NOS
Beppu 2015 [25]	1	1	1	1	1	1	1	1	8
Chung 2017 [16]	1	1	1	1	1	1	0	1	7
Markovina 2017 [15]	1	1	1	1	2	1	1	1	9
Hoendervangers 2018 [21]	1	1	1	1	1	1	0	1	7
Hoendervangers 2020 [23]	1	1	1	1	1	1	0	1	7
Thakur 2020 [27]	1	1	1	1	1	1	0	1	7
Chapman 2022 [31]	1	1	1	1	1	1	1	1	8

**Table 3:** Summary of the outcomes from the including studies.

Study	Intervention	pCR Rate	Downstaging Rate	R0 Resection Rate	Sphincter Preservation	OS	DFS
Latkauskas 2016 [13]	SCRT	4.4	30.9	86.5	70.3	78.0 (3-year)	59.0 (3-year)
	CRT	11.1	37.5	91.3	69.6	82.4 (3-year)	75.1 (3-year)
Kairevičė 2017 [20]	SCRT					62 (5-year)	45 (5-year)
	CRT					79 (5-year)	67 (5-year)
Bujko 2016 [14]	SCRT	16	NA	77	43	73 (3-year)	53 (3-year)
	CRT	12	NA	71	39	65 (3-year)	52 (3-year)
Ciseł 2019 [24]	SCRT					49 (8-year)	43 (8-year)
	CRT					49 (8-year)	41 (8-year)
Beppu 2015 [25]	SCRT	4.8	37.5	NA	93.3	95.1 (3-year)	83.8 (3-year)
	CRT	8.2	37.7	NA	85.2	93.1 (3-year)	73.8 (3-year)
Chung 2017 [16]	SCRT	21.1	47.4	NA	89.5	90 (2-year)	93.8 (2-year)
	CRT	13.2	26.4	NA	94.3	91.2 (2-year)	74.0 (2-year)
Markovina 2017 [15]	SCRT	28	75	NA	75.4	96 (3-year)	85 (3-year) *
	CRT	16	41	NA	72.5	88 (3-year)	68 (3-year)
Chapman 2022 [31]	SCRT	26.2	NA	94.2	72.3	NA	NA
	CRT	17.3	NA	89.8	60.6	NA	NA
Hoendervangers 2018 [21]	SCRT	6.4	46.8	NA	42.5	NA	NA
	CRT	16.2	56.1	NA	51.7	NA	NA
Xiao 2018 [22]	SCRT	7.14	21.43	NA	NA	NA	NA
	CRT	11.22	25.51	NA	NA	NA	NA
Aghili 2020 [26]	SCRT	32.3	80.8	100	100	NA	NA
	CRT	23.1	84.6	96.2	96.2	NA	NA
Hoendervangers 2020 [23]	SCRT	7.7	NA	91.9	NA	NA	NA
	CRT	12.6	NA	89	NA	NA	NA
Thakur 2020 [27]	SCRT	6.7	35.7	92.8	75	NA	NA
	CRT	0	53.8	92.3	62.5	NA	NA
Bahadoer 2020 [29]	SCRT	28	NA	90	63.6	89.1 (3-year)	23.7 (3-year DRTF)
	CRT	14	NA	90	58.8	88.8 (3-year)	30.4 (3-year DRTF)
Chakrabarti 2021 [30]	SCRT	13.3	75.4	100	65	NA	NA
	CRT	10.9	74.6	100	59.4	NA	NA
Jin 2022 [32]	SCRT	16.6	NA	91.5	52.8	86.5 (3-year)	64.5 (3-year)
	CRT	11.7	NA	87.8	56.1	75.1 (3-year)	62.3 (3-year)

* DRTF: first occurrence of locoregional failure, distant metastasis, new primary colorectal tumor, or treatment-related death. NA: not avalible.

## Supplementary Materials

The following supporting information can be downloaded at: https://www.mdpi.com/article/10.3390/curroncol29050297/s1. Figure S1. Forest plot of down staging rate after neoadjuvant treatment. Figure S2. Forest plot of ypT3-4 rate after neoadjuvant treatment. Figure S3. Forest plot of ypN0 rate after neoadjuvant treatment. Figure S4. Forest plot of the rate of local recurrence. Figure S5. Forest plot of the rate of distant metastasis. Figure S6. Forest plot of the R0 resection rate. Figure S7. Forest plot of the negative circumferential resection margin (CRM) rate. Figure S8. Forest plot for the rate of postoperative complications. Figure S9. Forest plot of the rate of acute toxicity. Figure S10. Forest plot of (A) the rate of late toxicity (B) the compliance of chemotherapy.
