# Supplementary material for: Neoadjuvant Short-Course Radiotherapy Followed by Consolidation Chemotherapy before Surgery for Treating Locally Advanced Rectal Cancer: A Systematic Review and Meta-Analysis
Source: Curr Oncol. 2022 May 19;29(5):3708–27. doi: 10.3390/curroncol29050297 (PMC9139840; doi:10.3390/curroncol29050297)
Supplement: Supplementary file 1 [file curroncol-29-00297-s001.zip › curroncol-1667825-supplementary.pdf]

Current Oncology

MDPI

Systematic Review

Neoadjuvant Short-Course Radiotherapy Followed by Consolidation Chemotherapy before Surgery for Treating Locally Advanced Rectal Cancer: A Systematic Review and Meta-Analysis

Chun-Kai Liao, Ya-Ting Kuo, Yueh-Chen Lin, Yih-Jong Chern, Yu-Jen Hsu, Yen-Lin Yu, Jy-Ming Chiang, Pao-Shiu Hsieh, Chien-Yuh Yeh and Jeng-Fu You

Supplementary Materials

Study or Subgroup	SCRT Events	SCRT Total	CRT Events	CRT Total	Weight	Risk Ratio M–H, Random, 95% CI
**7.1.1 SCRT no CCT vs. CRT**						
Hoendervangers 2018	182	391	2079	3659	20.1%	0.82 [0.73, 0.91]
Latkauskas 2016	21	68	27	72	8.8%	0.82 [0.52, 1.31]
Xiao 2018	21	98	25	98	7.8%	0.84 [0.51, 1.40]
**Subtotal (95% CI)**		**557**		**3829**	**36.7%**	**0.82 [0.74, 0.91]**
Total events	224		2131			
Heterogeneity: $Tau^2 = 0.00$; $Chi^2 = 0.01$, df = 2 (P = 1.00); $I^2 = 0\%$						
Test for overall effect: Z = 3.71 (P = 0.0002)						
**7.1.2 SCRT + CCT vs. CRT**						
Aghili 2020	25	31	22	26	15.6%	0.95 [0.75, 1.21]
Beppu 2015	28	104	30	61	10.2%	0.55 [0.36, 0.82]
Chakrabarti 2021	52	69	53	71	17.4%	1.01 [0.83, 1.22]
Chapman 2022	49	187	39	226	11.1%	1.52 [1.05, 2.21]
Chung 2017	9	19	14	53	5.5%	1.79 [0.93, 3.45]
Thakur 2020	5	14	7	13	3.5%	0.66 [0.28, 1.57]
**Subtotal (95% CI)**		**424**		**450**	**63.3%**	**1.00 [0.75, 1.32]**
Total events	168		165			
Heterogeneity: $Tau^2 = 0.08$; $Chi^2 = 17.56$, df = 5 (P = 0.004); $I^2 = 72\%$						
Test for overall effect: Z = 0.02 (P = 0.98)						
**Total (95% CI)**		**981**		**4279**	**100.0%**	**0.93 [0.78, 1.11]**
Total events	392		2296			
Heterogeneity: $Tau^2 = 0.04$; $Chi^2 = 22.64$, df = 8 (P = 0.004); $I^2 = 65\%$						
Test for overall effect: Z = 0.81 (P = 0.42)						
Test for subgroup differences: $Chi^2 = 1.64$, df = 1 (P = 0.20), $I^2 = 39.0\%$						

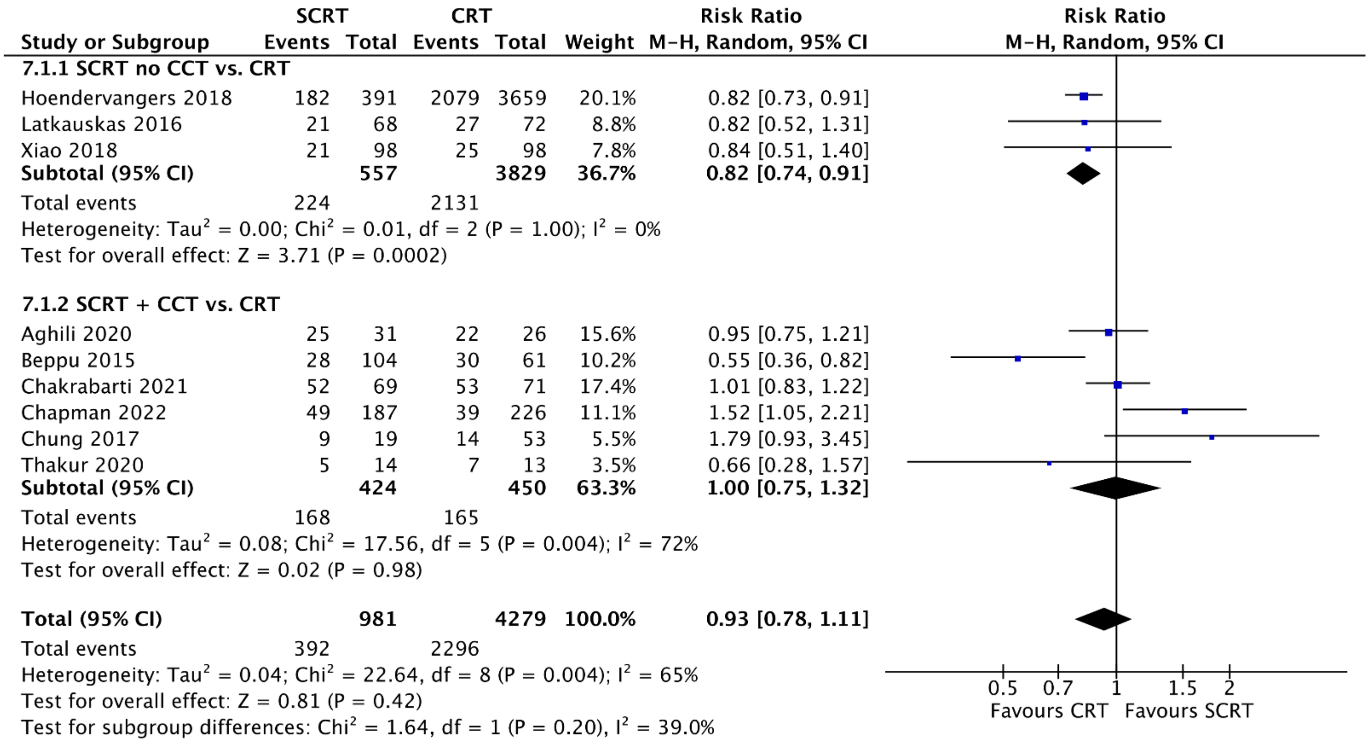

Figure S1. Forest plot of down staging rate after neoadjuvant treatment.

Study or Subgroup	SCRT Events	SCRT Total	CRT Events	CRT Total	Weight	Risk Ratio M-H, Random, 95% CI	Year
**8.1.1 SCRT no CCT vs. CRT**							
Latkauskas 2016	44	68	40	72	9.3%	1.16 [0.89, 1.53]	2016
Hoendervangers 2018	238	391	1764	3659	13.2%	1.26 [1.16, 1.38]	2018
Xiao 2018	36	98	42	98	7.7%	0.86 [0.61, 1.21]	2018
Hoendervangers 2020	147	246	132	246	12.0%	1.11 [0.95, 1.30]	2020
**Subtotal (95% CI)**		**803**		**4075**	**42.3%**	**1.15 [1.01, 1.31]**	
Total events	465		1978				
Heterogeneity: $Tau^2 = 0.01$; $Chi^2 = 6.00$, df = 3 (P = 0.11); $I^2 = 50\%$							
Test for overall effect: Z = 2.13 (P = 0.03)							
**8.1.2 SCRT + CCT vs. CRT**							
Beppu 2015	65	104	38	61	9.9%	1.00 [0.78, 1.28]	2015
Bujko 2016	133	220	123	205	12.0%	1.01 [0.86, 1.18]	2016
Markovina 2017	18	69	43	69	6.1%	0.42 [0.27, 0.65]	2017
Aghili 2020	13	31	12	26	4.2%	0.91 [0.51, 1.63]	2020
Bahadoer 2020	193	423	215	398	12.3%	0.84 [0.74, 0.97]	2020
Chakrabarti 2021	6	60	7	64	1.7%	0.91 [0.33, 2.57]	2021
Jin 2022	113	235	123	230	11.4%	0.90 [0.75, 1.08]	2022
**Subtotal (95% CI)**		**1142**		**1053**	**57.7%**	**0.87 [0.74, 1.01]**	
Total events	541		561				
Heterogeneity: $Tau^2 = 0.02$; $Chi^2 = 15.64$, df = 6 (P = 0.02); $I^2 = 62\%$							
Test for overall effect: Z = 1.80 (P = 0.07)							
**Total (95% CI)**		**1945**		**5128**	**100.0%**	**0.96 [0.83, 1.11]**	
Total events	1006		2539				
Heterogeneity: $Tau^2 = 0.04$; $Chi^2 = 51.13$, df = 10 (P < 0.00001); $I^2 = 80\%$							
Test for overall effect: Z = 0.60 (P = 0.55)							
Test for subgroup differences: $Chi^2 = 7.54$, df = 1 (P = 0.006), $I^2 = 86.7\%$							

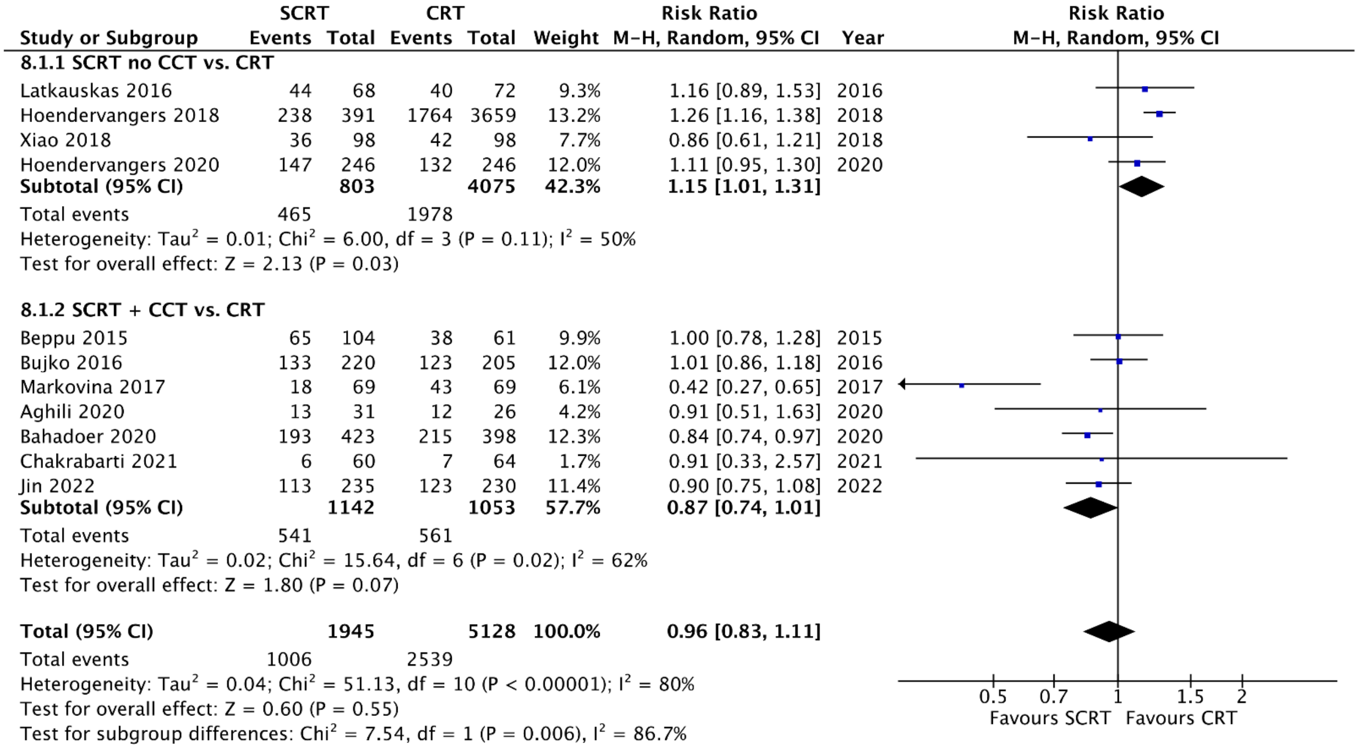

Figure S2. Forest plot of ypT3-4 rate after neoadjuvant treatment.

Study or Subgroup	SCRT Events	SCRT Total	CRT Events	CRT Total	Weight	Risk Ratio M-H, Random, 95% CI	Year
**9.1.1 SCRT no CCT vs. CRT**							
Latkauskas 2016	43	68	54	72	6.5%	0.84 [0.67, 1.06]	2016
Hoendervangers 2018	219	391	2454	3659	12.0%	0.84 [0.76, 0.91]	2018
Xiao 2018	62	98	74	98	7.8%	0.84 [0.69, 1.01]	2018
Hoendervangers 2020	155	246	171	246	10.4%	0.91 [0.80, 1.03]	2020
**Subtotal (95% CI)**		**803**		**4075**	**36.8%**	**0.85 [0.80, 0.91]**	
Total events	479		2753				
Heterogeneity: $Tau^2 = 0.00$; $Chi^2 = 1.15$, df = 3 (P = 0.77); $I^2 = 0\%$							
Test for overall effect: Z = 4.68 (P < 0.00001)							
**9.1.2 SCRT + CCT vs. CRT**							
Beppu 2015	77	104	41	61	7.1%	1.10 [0.89, 1.36]	2015
Bujko 2016	151	220	141	205	10.3%	1.00 [0.88, 1.13]	2016
Markovina 2017	51	69	40	69	5.9%	1.27 [1.00, 1.63]	2017
Bahadoer 2020	317	423	273	398	12.2%	1.09 [1.00, 1.19]	2020
Aghili 2020	27	31	23	26	7.6%	0.98 [0.81, 1.20]	2020
Chakrabarti 2021	52	60	52	64	9.2%	1.07 [0.91, 1.24]	2021
Jin 2022	168	235	160	230	10.8%	1.03 [0.91, 1.16]	2022
**Subtotal (95% CI)**		**1142**		**1053**	**63.2%**	**1.06 [1.01, 1.12]**	
Total events	843		730				
Heterogeneity: $Tau^2 = 0.00$; $Chi^2 = 4.48$, df = 6 (P = 0.61); $I^2 = 0\%$							
Test for overall effect: Z = 2.25 (P = 0.02)							
**Total (95% CI)**		**1945**		**5128**	**100.0%**	**0.98 [0.91, 1.06]**	
Total events	1322		3483				
Heterogeneity: $Tau^2 = 0.01$; $Chi^2 = 32.31$, df = 10 (P = 0.0004); $I^2 = 69\%$							
Test for overall effect: Z = 0.43 (P = 0.66)							
Test for subgroup differences: $Chi^2 = 25.75$, df = 1 (P < 0.00001), $I^2 = 96.1\%$							

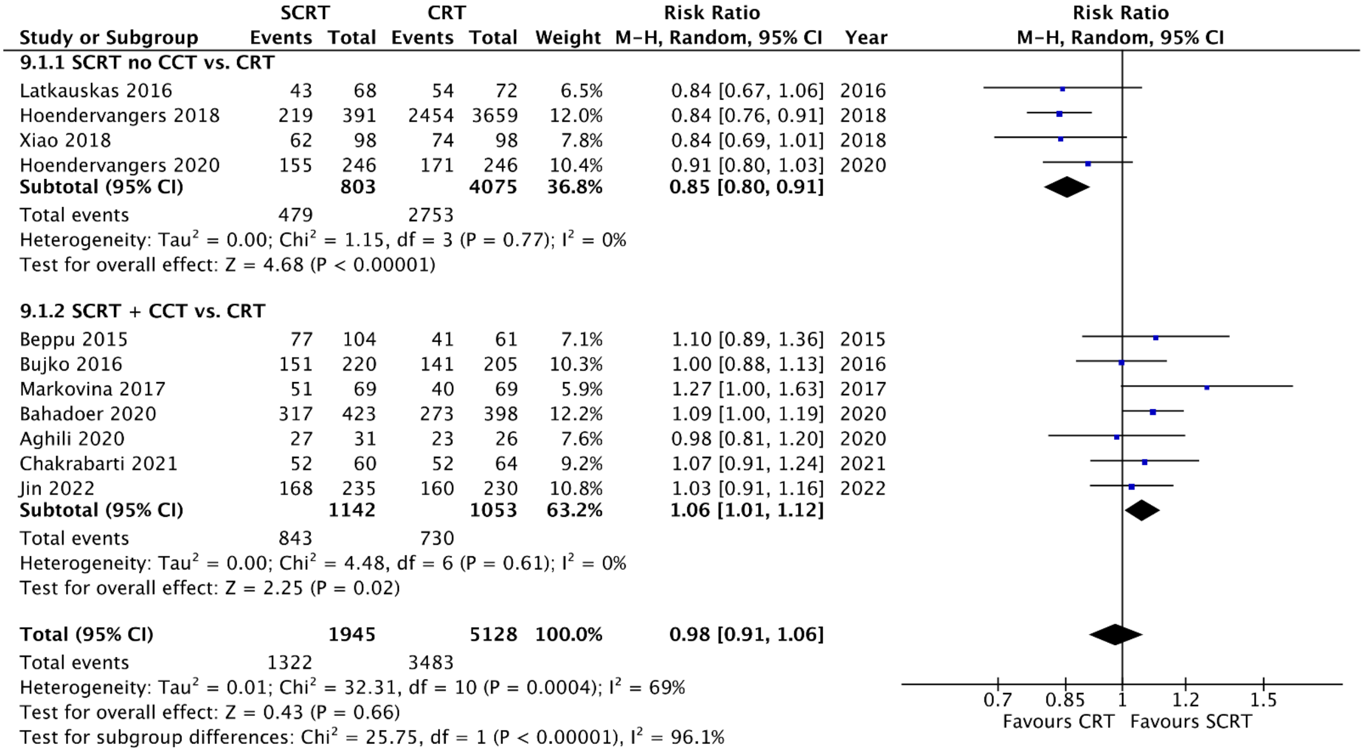

Figure S3. Forest plot of ypN0 rate after neoadjuvant treatment.

Study or Subgroup	SCRT Events	SCRT Total	CRT Events	CRT Total	Weight	Risk Ratio M-H, Random, 95% CI	Year
**10.1.1 SCRT no CCT vs. CRT**							
Kairevičė 2017	4	68	5	72	2.5%	0.85 [0.24, 3.02]	2017
**Subtotal (95% CI)**		**68**		**72**	**2.5%**	**0.85 [0.24, 3.02]**	
Total events	4		5				
Heterogeneity: Not applicable							
Test for overall effect: Z = 0.26 (P = 0.80)							
**10.1.2 SCRT + CCT vs. CRT**							
Beppu 2015	8	104	4	61	3.0%	1.17 [0.37, 3.73]	2015
Chung 2017	1	19	1	53	0.5%	2.79 [0.18, 42.42]	2017
Ciseł 2019	89	261	80	254	66.3%	1.08 [0.85, 1.39]	2019
Thakur 2020	2	14	1	13	0.8%	1.86 [0.19, 18.13]	2020
Bahadoer 2020	29	462	17	450	11.9%	1.66 [0.93, 2.98]	2020
Jin 2022	20	302	23	297	12.2%	0.86 [0.48, 1.52]	2022
Chapman 2022	5	187	5	226	2.7%	1.21 [0.36, 4.11]	2022
**Subtotal (95% CI)**		**1349**		**1354**	**97.5%**	**1.12 [0.92, 1.38]**	
Total events	154		131				
Heterogeneity: $Tau^2 = 0.00$; $Chi^2 = 3.31$, df = 6 (P = 0.77); $I^2 = 0\%$							
Test for overall effect: Z = 1.13 (P = 0.26)							
**Total (95% CI)**		**1417**		**1426**	**100.0%**	**1.12 [0.91, 1.37]**	
Total events	158		136				
Heterogeneity: $Tau^2 = 0.00$; $Chi^2 = 3.50$, df = 7 (P = 0.84); $I^2 = 0\%$							
Test for overall effect: Z = 1.07 (P = 0.28)							
Test for subgroup differences: $Chi^2 = 0.19$, df = 1 (P = 0.67), $I^2 = 0\%$							

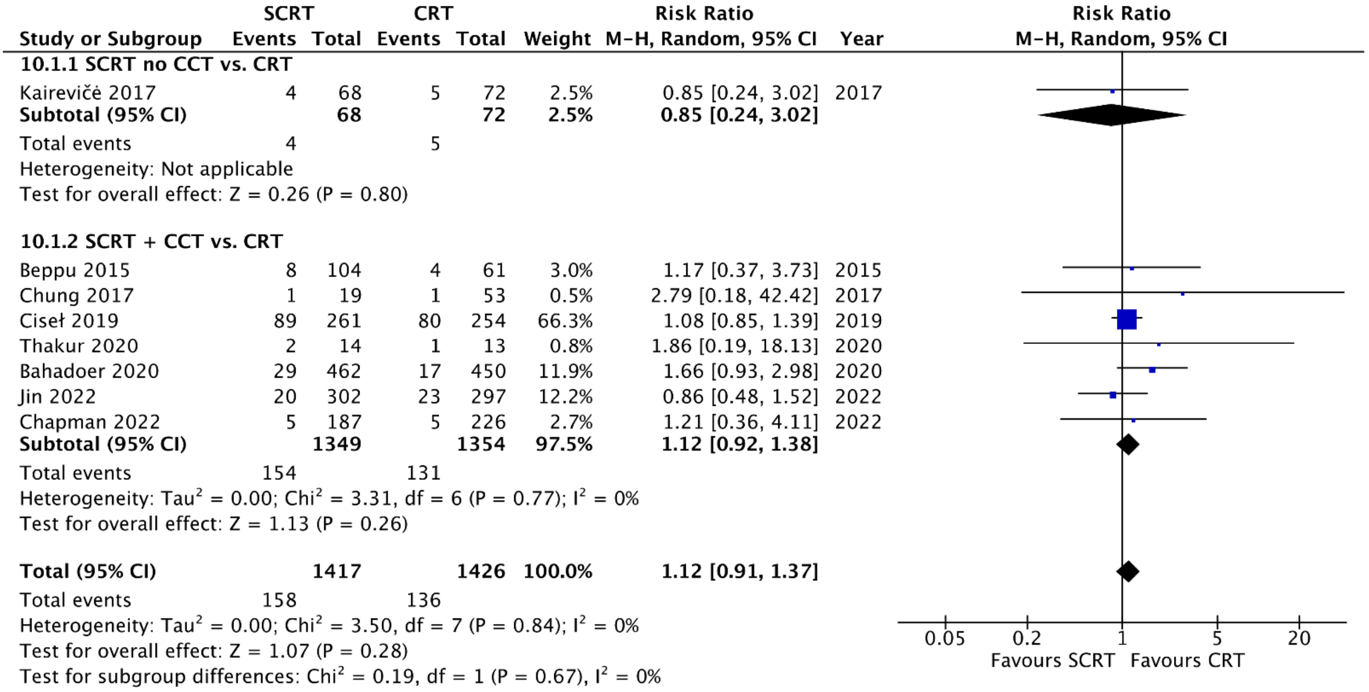

Figure S4. Forest plot of the rate of local recurrence.

Study or Subgroup	SCRT Events	SCRT Total	CRT Events	CRT Total	Weight	Risk Ratio M-H, Random, 95% CI	Year
**11.1.1 SCRT no CCT vs. CRT**							
Kairevičė 2017	21	68	16	72	11.5%	1.39 [0.79, 2.43]	2017
**Subtotal (95% CI)**		**68**		**72**	**11.5%**	**1.39 [0.79, 2.43]**	
Total events	21		16				
Heterogeneity: Not applicable							
Test for overall effect: Z = 1.15 (P = 0.25)							
**11.1.2 SCRT + CCT vs. CRT**							
Chung 2017	1	19	12	53	1.3%	0.23 [0.03, 1.67]	2017
Ciseł 2019	91	261	83	254	26.0%	1.07 [0.84, 1.36]	2019
Thakur 2020	1	14	1	13	0.7%	0.93 [0.06, 13.37]	2020
Bahadoer 2020	86	462	123	450	25.9%	0.68 [0.53, 0.87]	2020
Chapman 2022	20	187	30	226	12.3%	0.81 [0.47, 1.37]	2022
Jin 2022	65	302	67	297	22.4%	0.95 [0.71, 1.29]	2022
**Subtotal (95% CI)**		**1245**		**1293**	**88.5%**	**0.86 [0.68, 1.07]**	
Total events	264		316				
Heterogeneity: $Tau^2 = 0.03$; $Chi^2 = 8.87$, df = 5 (P = 0.11); $I^2 = 44\%$							
Test for overall effect: Z = 1.34 (P = 0.18)							
**Total (95% CI)**		**1313**		**1365**	**100.0%**	**0.90 [0.72, 1.14]**	
Total events	285		332				
Heterogeneity: $Tau^2 = 0.04$; $Chi^2 = 11.45$, df = 6 (P = 0.08); $I^2 = 48\%$							
Test for overall effect: Z = 0.87 (P = 0.38)							
Test for subgroup differences: $Chi^2 = 2.46$, df = 1 (P = 0.12), $I^2 = 59.4\%$							

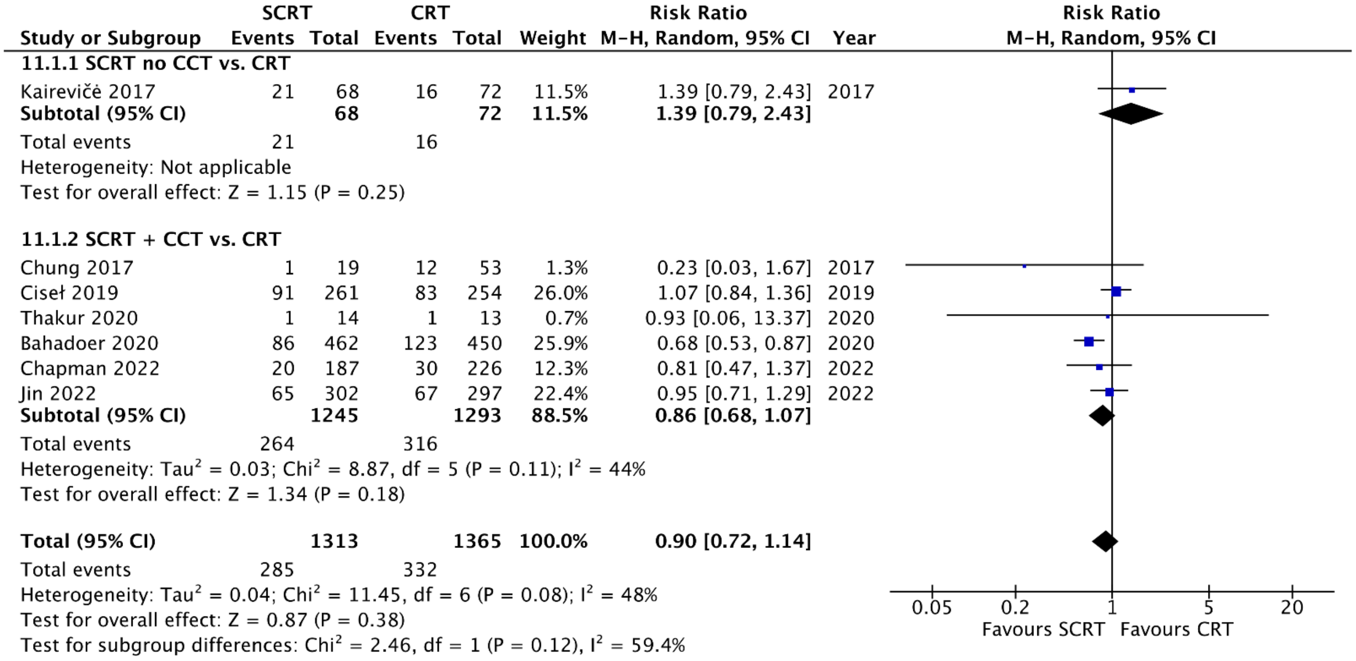

Figure S5. Forest plot of the rate of distant metastasis.

Study or Subgroup	SCRT Events	SCRT Total	CRT Events	CRT Total	Weight	Risk Ratio M-H, Random, 95% CI	Year
**12.1.1 SCRT no CCT vs. CRT**							
Latkauskas 2016	57	68	64	72	2.4%	0.94 [0.83, 1.08]	2016
Hoendervangers 2020	226	246	219	246	11.9%	1.03 [0.97, 1.09]	2020
**Subtotal (95% CI)**		**314**		**318**	**14.3%**	**1.01 [0.93, 1.09]**	
Total events	283		283				
Heterogeneity: Tau^2 = 0.00; Chi^2 = 1.53, df = 1 (P = 0.22); I^2 = 35%							
Test for overall effect: Z = 0.16 (P = 0.87)							
**12.1.2 SCRT + CCT vs. CRT**							
Bujko 2016	202	261	178	254	3.8%	1.10 [1.00, 1.23]	2016
Chung 2017	17	19	47	53	1.3%	1.01 [0.84, 1.21]	2017
Thakur 2020	7	8	7	8	0.3%	1.00 [0.69, 1.45]	2020
Aghili 2020	31	31	25	26	4.0%	1.04 [0.94, 1.15]	2020
Bahadoer 2020	382	423	360	398	19.0%	1.00 [0.95, 1.04]	2020
Chakrabarti 2021	60	60	64	64	35.2%	1.00 [0.97, 1.03]	2021
Chapman 2022	213	226	168	187	11.8%	1.05 [0.99, 1.11]	2022
Jin 2022	215	235	202	230	10.4%	1.04 [0.98, 1.11]	2022
**Subtotal (95% CI)**		**1263**		**1220**	**85.7%**	**1.02 [0.99, 1.05]**	
Total events	1127		1051				
Heterogeneity: Tau^2 = 0.00; Chi^2 = 9.03, df = 7 (P = 0.25); I^2 = 22%							
Test for overall effect: Z = 1.59 (P = 0.11)							
**Total (95% CI)**		**1577**		**1538**	**100.0%**	**1.02 [1.00, 1.04]**	
Total events	1410		1334				
Heterogeneity: Tau^2 = 0.00; Chi^2 = 9.45, df = 9 (P = 0.40); I^2 = 5%							
Test for overall effect: Z = 1.67 (P = 0.09)							
Test for subgroup differences: Chi^2 = 0.12, df = 1 (P = 0.72), I^2 = 0%							

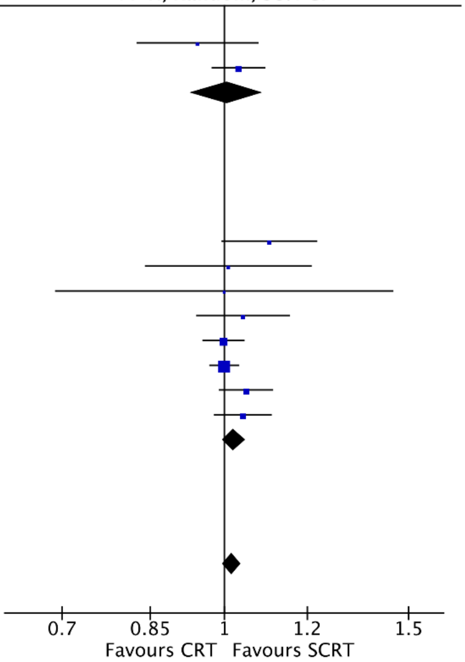

Figure S6. Forest plot of the R0 resection rate.

Study or Subgroup	SCRT Events	SCRT Total	CRT Events	CRT Total	Weight	Risk Ratio M-H, Random, 95% CI	Year
**13.1.1 SCRT no CCT vs. CRT**							
Latkauskas 2016	57	68	64	72	2.0%	0.94 [0.83, 1.08]	2016
Xiao 2018	94	98	95	98	11.9%	0.99 [0.94, 1.04]	2018
Hoendervangers 2020	226	246	219	246	10.4%	1.03 [0.97, 1.09]	2020
**Subtotal (95% CI)**		**412**		**416**	**24.3%**	**1.00 [0.97, 1.04]**	
Total events	377		378				
Heterogeneity: Tau^2 = 0.00; Chi^2 = 2.04, df = 2 (P = 0.36); I^2 = 2%							
Test for overall effect: Z = 0.18 (P = 0.86)							
**13.1.2 SCRT + CCT vs. CRT**							
Beppu 2015	99	104	57	61	5.5%	1.02 [0.94, 1.10]	2015
Bujko 2016	36	38	34	40	1.5%	1.11 [0.96, 1.30]	2016
Chung 2017	17	19	47	53	1.0%	1.01 [0.84, 1.21]	2017
Xiao 2018	94	98	95	98	11.9%	0.99 [0.94, 1.04]	2018
Ciseł 2019	137	220	134	205	1.7%	0.95 [0.83, 1.10]	2019
Bahadoer 2020	385	423	363	398	18.9%	1.00 [0.96, 1.04]	2020
Chakrabarti 2021	60	60	64	64	35.2%	1.00 [0.97, 1.03]	2021
**Subtotal (95% CI)**		**962**		**919**	**75.7%**	**1.00 [0.98, 1.02]**	
Total events	828		794				
Heterogeneity: Tau^2 = 0.00; Chi^2 = 2.96, df = 6 (P = 0.81); I^2 = 0%							
Test for overall effect: Z = 0.04 (P = 0.97)							
**Total (95% CI)**		**1374**		**1335**	**100.0%**	**1.00 [0.98, 1.02]**	
Total events	1205		1172				
Heterogeneity: Tau^2 = 0.00; Chi^2 = 4.88, df = 9 (P = 0.84); I^2 = 0%							
Test for overall effect: Z = 0.12 (P = 0.90)							
Test for subgroup differences: Chi^2 = 0.02, df = 1 (P = 0.89), I^2 = 0%							

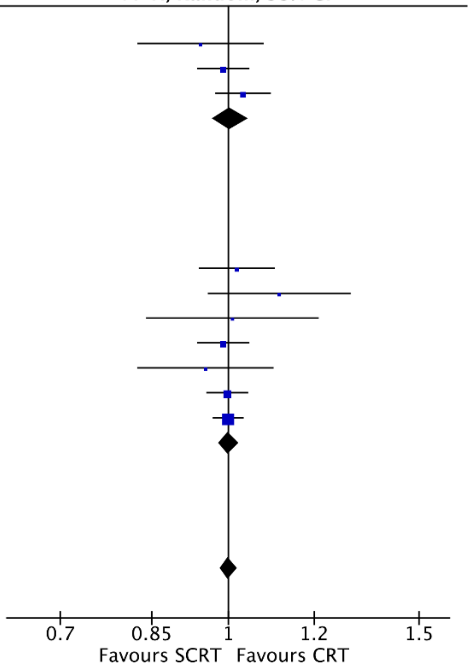

Figure S7. Forest plot of the negative circumferential resection margin (CRM) rate.

Study or Subgroup	SCRT Events	SCRT Total	CRT Events	CRT Total	Weight	Risk Ratio M-H, Random, 95% CI	Year
**17.1.1 SCRT no CCT vs. CRT**							
Latkauskas 2016	24	68	19	72	3.7%	1.34 [0.81, 2.21]	2016
Xiao 2018	8	98	9	98	1.1%	0.89 [0.36, 2.21]	2018
Hoendervangers 2020	102	246	93	246	19.7%	1.10 [0.88, 1.36]	2020
**Subtotal (95% CI)**		**412**		**416**	**24.6%**	**1.12 [0.92, 1.36]**	
Total events	134		121				
Heterogeneity: $Tau^2 = 0.00$; $Chi^2 = 0.76$, df = 2 (P = 0.68); $I^2 = 0\%$							
Test for overall effect: Z = 1.13 (P = 0.26)							
**17.1.2 SCRT + CCT vs. CRT**							
Beppu 2015	7	104	3	61	0.5%	1.37 [0.37, 5.10]	2015
Chung 2017	5	19	7	53	0.9%	1.99 [0.72, 5.53]	2017
Markovina 2017	32	69	30	69	6.9%	1.07 [0.74, 1.54]	2017
Ciseł 2019	62	220	49	205	9.0%	1.18 [0.85, 1.63]	2019
van der Valk 2020	215	426	189	400	48.1%	1.07 [0.93, 1.23]	2020
Aghili 2020	6	31	3	26	0.6%	1.68 [0.46, 6.06]	2020
Chakrabarti 2021	25	60	21	64	4.4%	1.27 [0.80, 2.01]	2021
Jin 2022	33	235	36	230	5.0%	0.90 [0.58, 1.39]	2022
**Subtotal (95% CI)**		**1164**		**1108**	**75.4%**	**1.09 [0.98, 1.22]**	
Total events	385		338				
Heterogeneity: $Tau^2 = 0.00$; $Chi^2 = 3.40$, df = 7 (P = 0.85); $I^2 = 0\%$							
Test for overall effect: Z = 1.56 (P = 0.12)							
**Total (95% CI)**		**1576**		**1524**	**100.0%**	**1.10 [1.00, 1.21]**	
Total events	519		459				
Heterogeneity: $Tau^2 = 0.00$; $Chi^2 = 4.20$, df = 10 (P = 0.94); $I^2 = 0\%$							
Test for overall effect: Z = 1.92 (P = 0.06)							
Test for subgroup differences: $Chi^2 = 0.04$, df = 1 (P = 0.84), $I^2 = 0\%$							

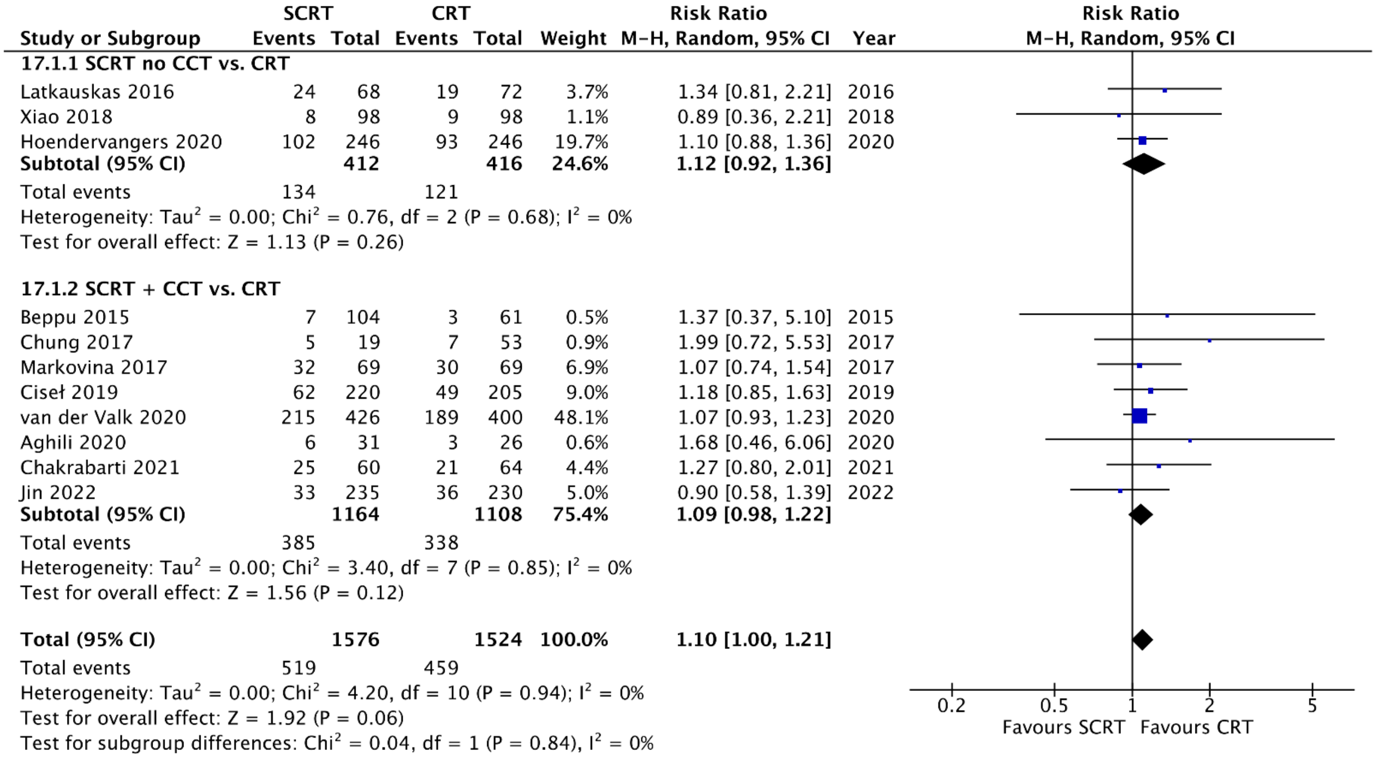

Figure S8. Forest plot for the rate of postoperative complications.

Study or Subgroup	SCRT Events	SCRT Total	CRT Events	CRT Total	Weight	Risk Ratio M-H, Random, 95% CI	Year
**18.1.1 SCRT no CCT vs. CRT**							
Xiao 2018	5	98	26	98	10.1%	0.19 [0.08, 0.48]	2018
**Subtotal (95% CI)**		**98**		**98**	**10.1%**	**0.19 [0.08, 0.48]**	
Total events	5		26				
Heterogeneity: Not applicable							
Test for overall effect: Z = 3.53 (P = 0.0004)							
**18.1.2 SCRT + CCT vs. CRT**							
Chung 2017	1	19	1	53	2.2%	2.79 [0.18, 42.42]	2017
Markovina 2017	26	69	22	69	15.3%	1.18 [0.75, 1.87]	2017
Ciseł 2019	62	256	61	259	16.9%	1.03 [0.76, 1.40]	2019
Thakur 2020	2	14	8	13	6.6%	0.23 [0.06, 0.90]	2020
Aghili 2020	8	33	6	27	10.0%	1.09 [0.43, 2.76]	2020
Bahadoer 2020	222	460	111	441	17.9%	1.92 [1.59, 2.31]	2020
Chakrabarti 2021	2	69	3	71	4.6%	0.69 [0.12, 3.98]	2021
Jin 2022	79	298	37	293	16.4%	2.10 [1.47, 3.00]	2022
**Subtotal (95% CI)**		**1218**		**1226**	**89.9%**	**1.30 [0.91, 1.85]**	
Total events	402		249				
Heterogeneity: $Tau^2 = 0.13$; $Chi^2 = 24.94$, df = 7 (P = 0.0008); $I^2 = 72\%$							
Test for overall effect: Z = 1.46 (P = 0.15)							
**Total (95% CI)**		**1316**		**1324**	**100.0%**	**1.02 [0.66, 1.57]**	
Total events	407		275				
Heterogeneity: $Tau^2 = 0.27$; $Chi^2 = 45.38$, df = 8 (P < 0.00001); $I^2 = 82\%$							
Test for overall effect: Z = 0.09 (P = 0.93)							
Test for subgroup differences: $Chi^2 = 14.58$, df = 1 (P = 0.0001), $I^2 = 93.1\%$							

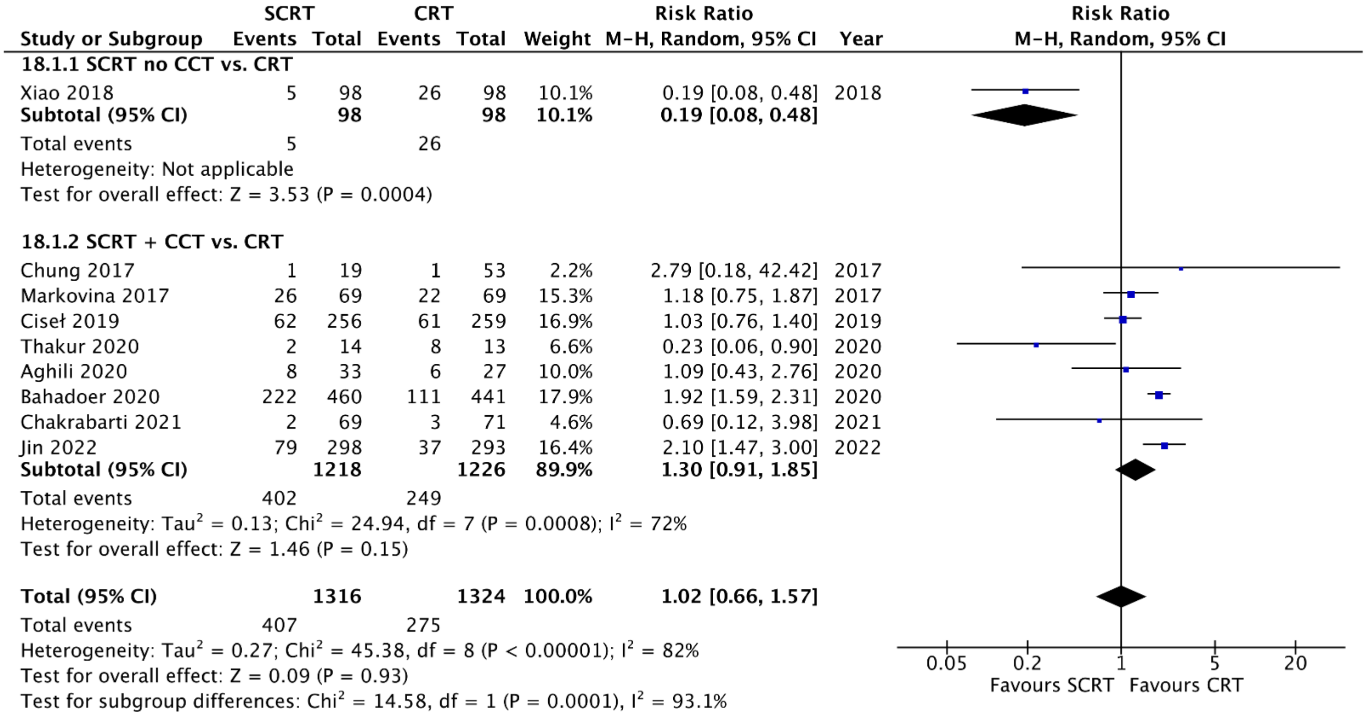

Figure S9. Forest plot of the rate of acute toxicity.

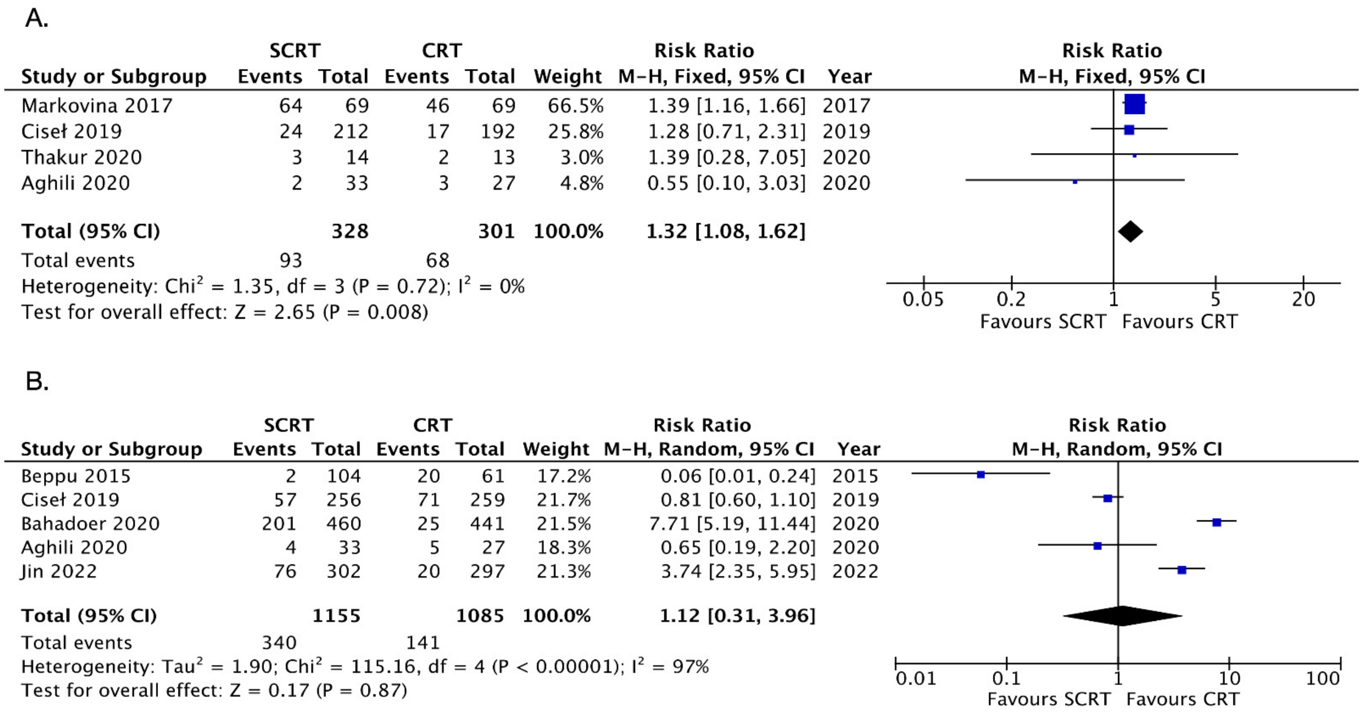

Figure S10. Forest plot of (**A**) the rate of late toxicity (**B**) the compliance of chemotherapy.
